# Behavioral intervention grounded in motivational interviewing and behavioral economics shows promise with Black and English-speaking Latino persons living with HIV with unsuppressed HIV viral load in New York City: A mixed methods pilot study

**DOI:** 10.3389/fpubh.2022.916224

**Published:** 2022-09-15

**Authors:** Marya Gwadz, Samantha Serrano, Sebastian Linnemayr, Charles M. Cleland, Sabrina R. Cluesman, Robin M. Freeman, Kinsey Kellam, Corey De Stefano, Khadija Israel, Emily Pan

**Affiliations:** ^1^Intervention Innovations Team Lab (IIT-Lab), New York University Silver School of Social Work, New York, NY, United States; ^2^Center for Drug Use and HIV Research, School of Global Public Health, New York University, New York, NY, United States; ^3^Pardee RAND Graduate School, Santa Monica, CA, United States; ^4^Department of Biostatistics, New York University School of Medicine, New York, NY, United States; ^5^Department of Population Health, New York University School of Medicine, New York, NY, United States; ^6^Independent Consultant, Brooklyn, NY, United States; ^7^North Jersey Community Research Initiative, Newark, NJ, United States; ^8^Rory Meyers School of Nursing, New York University, New York, NY, United States

**Keywords:** HIV care continuum, racial/ethnic disparities, behavioral economics, intervention, HIV viral suppression, text message, conditional economic incentives, motivational interviewing

## Abstract

**Introduction:**

Sustained HIV viral suppression is the ultimate goal of HIV treatment. African American/Black and Latino persons with HIV (PWH) in the United States are less likely than their White peers to achieve and sustain viral suppression. To address these disparities, we developed a “low-touch” behavioral intervention drawing on motivational interviewing and behavioral economics. The intervention had three main components: (1) a motivational interviewing counseling session, (2) 16 weeks of automated text messages and quiz questions about HIV management, where participants earned points by answering quiz questions, and 3) a lottery prize, based on viral suppression status, number of points earned, and chance (max. $275).

**Materials and methods:**

The intervention was tested in a pre-test/post-test design. The present pilot study used mixed methods to explore the intervention's feasibility, acceptability, impact, and ways it could be improved. Participants engaged in a baseline assessment, qualitative interview, and two structured follow-up assessments over an 8-month period, and provided laboratory reports to document HIV viral load. We carried out descriptive quantitative analyses. Qualitative data were analyzed using a directed content analysis approach. Data integration was carried out using the joint display method.

**Findings:**

Participants (*N* = 40) were 50 years old, on average (SD = 11), and approximately half (58%) were male. Close to two-thirds (68%) were African American/Black and 32% were Latino. Participants were diagnosed with HIV 22 years ago on average (SD = 8). The intervention was feasible (e.g., mean number of quiz questions answered = 13/16) and highly acceptable. While not powered to assess efficacy, the proportion with suppressed HIV viral load increased from baseline to follow-up (46% participants at the first, 52% participants at the second follow-up evidenced HIV viral suppression). In qualitative analyses, perspectives included that overall, the intervention was acceptable and useful, it was distinct from other programs, lottery prizes were interesting and appreciated but not sufficient to motivate behavior change, and the structure of lottery prizes was not sufficiently clear. Regarding data integration, qualitative data shed light on and extended quantitative results, and added richness and context.

**Conclusion:**

This low-touch intervention approach is sufficiently promising to warrant refinement and study in future research.

## Introduction

The public health system in the United States is oriented toward supporting persons with HIV (PWH) in achieving HIV viral suppression (1). This is because those with HIV viral suppression have a higher quality of life (2), lower morbidity (3), fewer hospitalizations (4, 5), longer lifespans (2), and virtually no chance of transmitting HIV to sexual partners (6). Yet, subpopulations of PWH, mainly those located in high-risk contexts, evidence serious barriers to consistent engagement along the HIV care continuum, a model comprised of steps toward HIV viral suppression that includes engagement in HIV care, medication uptake, and high levels of medication adherence (7–10). African American/Black and Latino persons are greatly over-represented in the population of PWH compared to their proportions in the underlying general population (11). Moreover, substantial racial/ethnic inequities persist in rates of engagement along the HIV care continuum, as well as in HIV-related morbidity and mortality rates (12). The larger public health goal of ending the HIV epidemic in the United States will not be achieved without reducing these racial/ethnic inequities (13).

While most PWH engage in HIV care and *achiev*e HIV viral suppression, approximately half of PWH do not *sustain* HIV viral suppression (14). Sustained HIV viral suppression is lowest among African American/Black and Latino individuals from low socio-economic status backgrounds (15): An estimated 41% of African American/Black PWH sustain viral suppression, compared to 50% among Latino and 56% among White PWH (15). Indeed, recent past research found starting and stopping HIV antiretroviral therapy is common among African American/Black and Latino PWH, including in response to life disruptions that occur in the context of severe poverty (e.g., lost housing or the end of an important relationship) (9).

These serious and persistent racial/ethnic inequities in engagement along the HIV care continuum and in HIV health outcomes signal the need for evolution and improvements in effective behavioral intervention approaches for PWH, including for those with the greatest barriers to HIV viral suppression. Our team's past research has shed light on such behavioral interventions to address the constellation of barriers that African American/Black and Latino PWH experience to engagement along the HIV care continuum, primarily taking counseling approaches (16–19). While the evidence base for the effectiveness and utility of counseling and other such behavioral interventions delivered by professional staff is robust (20, 21), as technology evolves and cell phone and smartphone use increases, researchers are increasingly turning to technology-facilitated interventions, including those that require little or no facilitation on the part of staff, called low-touch interventions (22–25). The present study extends this past research and explores a new relatively “low-touch” intervention comprised mainly of components that are either automated or that do not require staff time to be administered, complemented by staff-facilitated components, grounded in the motivational interviewing counseling approach and behavioral economics (26, 27).

Motivational interviewing is an evidence-based directive and collaborative counseling approach for behavior change that elicits participants' values, perspectives, and questions, identifies ambivalence and discrepancies, and corrects misinformation with permission, to thereby foster durable intrinsic motivation and readiness for change (28, 29). Across a range of health outcomes, motivational interviewing interventions have been found effective at clinically significant levels (30–32). Motivational interviewing has been found to be particularly effective with African American/Black and Latino populations compared to White populations (30). This may be because autonomy is often not supported in programs directed at African American/Black and Latino populations, and at the same time health beliefs and emotions such as distrust and fear commonly impede behavior change (18, 33, 34). Thus, aspects of motivational interviewing such as respect for individuals' views on the causes and consequences of health concerns, non-coercion, a focus on strengths, non-judgment, and autonomy support may resonate and have utility in this context (18, 33, 34).

Behavioral economics combines insights from the fields of psychology and economics to explain human decision making, with a focus on decisions and behaviors that might be deemed irrational in some frameworks including classic economic theories, such as the decision to decline to take HIV antiretroviral therapy, even in the face of adverse health consequences (35). Behavioral economics demonstrates that people rarely simply weigh the benefits and drawbacks of an action and then choose the best possible option. Instead, individuals are typically influenced by emotion or innate bias, such as an over-emphasis on the present combined with a lack of attention to future consequences of one's actions. Thus, people commonly make choices and engage in behaviors that may not be considered in their best interests in the long term (26, 27).

To circumvent biases, behavioral economics uses rewards and/or “nudges” to alter behavior. For example, participants may have the opportunity to win a prize if a behavior change goal is achieved, called a conditional economic incentive. Nudges are subtle and often indirect reminders that attempt to influence behavior through the way choices are made, taking into consideration behavioral biases. Prize drawings leverage the cognitive bias of overestimating small probabilities (leading individuals to participate in the prize drawing because they overestimate their chance of winning) and also increase salience (prizes keep a behavior high on a person's mental priority list) (27). Thus, extrinsic rewards such as conditional economic incentives in the form of prizes can be used to enhance intrinsic motivation and support the development of long-term habits, based on the assumption that motivation for health and wellbeing is universal, and therefore that behavior change is possible when one's values, goals, and motivation align with the incentivized behavior. In practice, behavioral economics is framed as a tool to support participants in their journey toward health. Importantly, these interventions generally require less staff time to implement than counseling-based interventions, and are therefore less costly, and can be often be carried out using mHealth, i.e., remotely (36). Indeed, efficient and low-cost behavioral interventions to increase HIV viral suppression are sorely needed but generally lacking (37), and the ongoing severe acute respiratory syndrome coronavirus 2 (SARS-CoV-2) pandemic highlights the need for remote services and technology-based health interventions (38).

Conditional economic incentives to improve HIV treatment adherence and support viral suppression is a relatively new area of study. Galarraga et al. carried out a substantive review of programs that used conditional economic incentives to improve HIV treatment adherence, mainly those carried out in clinical settings, including interventions grounded in behavioral economics (39). They found that when appropriately implemented, conditional economic incentives can help PWH improve their adherence to HIV treatment in the short-term, while incentives are in place. However, they noted mechanisms to increase habit formation or maintenance effects in the longer-term warrant more investigation. The review further highlights the potential of concepts from self-determination theory to bolster conditional economic incentive interventions. El-Sadr et al. (40) carried out a trial of a conditional economic incentive intervention with PWH with and without HIV viral suppression (HPTN 065) and found modest effects: The overall proportion of patients with viral suppression was 3.8% higher (95%CI, 0.7–6.8%) at financial incentive sites compared with standard of care sites. The present study extends this past research, focusing on PWH recruited from the community who may not be well-engaged in HIV care and who did not evidence HIV viral suppression, and is grounded, in part, in self-determination theory as we describe below.

Linnemayr et al. created an HIV prevention intervention grounded in behavioral economics called Mobile Technology and Incentives (MOTIVES) (27). MOTIVES used text messages (TMs) in combination with behavioral economic incentives to improve the retention of HIV prevention information and increase the frequency of HIV testing among Latino/a men who have sex with men and transgender women. Participants assigned to the intervention arm of the MOTIVES trial received TMs with HIV prevention information plus quiz questions (QQs) that provided the possibility of winning prizes. The study found incentives affected participants' HIV testing frequency where the frequency of HIV testing was higher in the intervention group relative to the comparison group. On average, 24.9% of participants in the arm that received text messages and quiz questions (TMQQs) tested for HIV within a given 3-month period, compared with 13.0% in the comparison group (41). The present study draws on the TMQQ method developed in the MOTIVES study (27), which we extended to and adapted for the population of African American/Black and Latino PWH with non-suppressed HIV viral load.

## Methods

### Overview

The present pilot study uses mixed methods in a convergent parallel design (42) to explore the acceptability and feasibility of a behavioral intervention called the Silver Community Action Project (SCAP), as well as participants' perspectives on the ways SCAP could be improved. The study was not powered for efficacy, but we also explored how motivation (the intervention's primary mechanism of action) and rates of HIV viral suppression at the two follow-up periods may have changed as potential evidence of non-futility of the intervention, and also elicited participants' perspectives on the utility of the intervention in qualitative research. The aim of the SCAP intervention was to increase rates of HIV viral suppression among African American/Black and Latino PWH with non-suppressed HIV viral load at enrollment. We used a pre-test/post-test design to evaluate the intervention's effects. Consistent with the convergent parallel mixed methods design, quantitative and qualitative elements were carried out in the same phase of the research process, methods were weighed equally, the two components were analyzed independently, and results were interpreted together (42). The study was carried out between 10/15/2019 and 4/1/2021 in New York City. We implemented study activities in-person at a project field site between 10/15/2019 and 3/12/2020. New York City was an early COVID-19 epicenter (43). On 3/12/2020, in-person activities with human subjects were suspended at our institution due to the COVID-19 pandemic. The study transitioned to a virtual format at that time. We enrolled 40 PWH with non-suppressed HIV viral load (> 200 copies/mL) and followed them over 8–10 months. The SCAP intervention (described in more detail below) had three main components: a counseling session grounded in motivational interviewing, 16 weeks of TMQQs during which participants could earn points for responding, and a lottery prize ranging from $5 to $275 based on three factors: whether HIV viral suppression was achieved, the number of points earned in the TMQQ component, and chance. Lottery prizes were allocated to participants by spinning a prize wheel. The prize wheel was similar to a roulette wheel. It was colorful and had 10 sections, where one section was clearly marked as triggering the biggest prize if the wheel stopped spinning at that section. The intervention's primary outcome was HIV viral suppression (HIV viral load ≤ 200 copies/mL) and we also assessed health-related quality of life. Structured follow-up assessments, including assessment of HIV viral load levels, were carried out at two time periods: the first follow-up assessment was carried out 4–6 months after baseline and the second at 7–10 months post-baseline. A check-in contact and brief qualitative interview were carried out with all participants at ~8-weeks post-baseline (half-way through the TMQQ component). The study was managed in the Research Electronic Data Capture (REDCap) platform. REDCap is a cloud-based platform for data capture designed for clinical research (44, 45). Assessments were programmed in REDCap and administered to participants by trained interviewers. Compensation was provided to participants using the Greenphire ClinCard system, a refillable debit card for research compensation. The study was approved by the Institutional Review Board at New York University and participants gave informed consent for study activities.

### Procedures to develop the SCAP intervention

**Figure 1:**
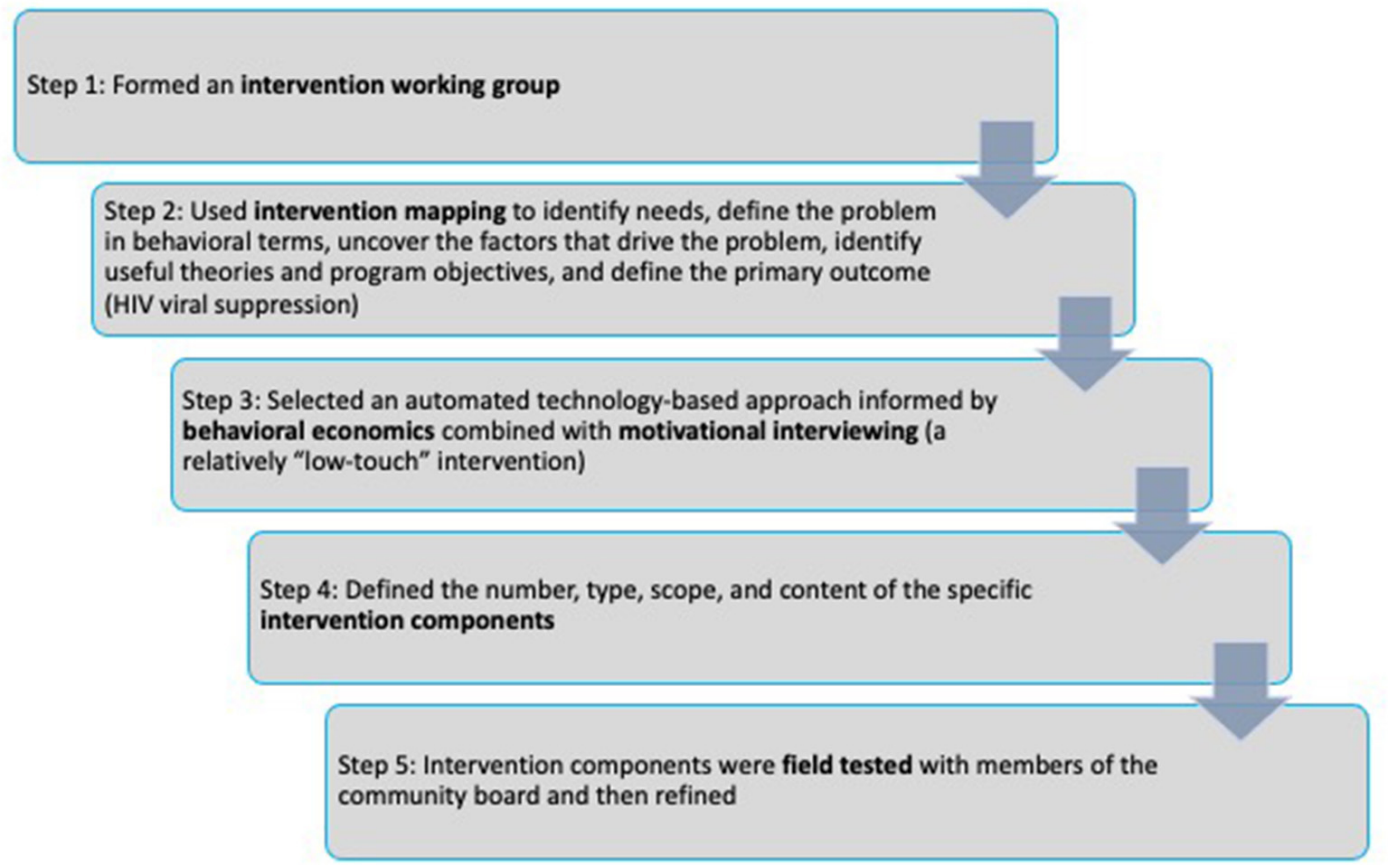
Schematic describing the steps taken to develop the SCAP intervention.

The SCAP intervention was based in part on the work of Linnemayr et al. (27) as described above, and our own research on counseling approaches for this population (9, 18, 33, 46). First, we established an intervention working group made up of members of our research team (which included Dr. Linnemayr) and a community advisory board comprised of members of the target population. As shown in Figure 1, in step 2, the intervention working group carried out an iterative intervention mapping process (47) that started with a needs assessment and review of the literature. The intervention working group then focused on the problem of non-suppressed HIV viral load, uncovered the factors that drive this problem, entertained a range of intervention objectives, and defined the primary outcome (increasing HIV viral suppression rates). In particular, the intervention working group focused on the need for intrinsic motivation and personal goals pertaining to HIV viral suppression as fundamental to behavior change, and the need for participants who wished to achieve viral suppression to develop durable habits to take HIV antiretroviral therapy over time. The intervention working group drew on theories of behavioral economics (described in the Introduction) and self-determination theory (16). Self-determination theory is a macro theory of human motivation and personality that concerns the innate and fundamental needs for autonomy (people need to feel in control of their own behaviors and goals), competence (people need to gain mastery of tasks and learn different skills), and connection or relatedness (people need to experience a sense of belonging and attachment to other people) (48). The most volitional and highest quality forms of motivation emerge when these three needs are supported by the larger environment (48).

In step 3, the intervention working group determined that low-touch, technology-based interventions and new approaches are needed for this population and this public health problem and that behavioral economic approaches hold promise, but that a purely automated intervention on its own may not be sufficient for this population and this problem. In step 4, the number, type, scope, and content of the specific intervention components were defined. The intervention components developed in the present study were designed to support participants' autonomy and build motivation for behavior change, both conscious and unconscious motivation. Motivational interviewing was selected as the main counseling approach (29) and motivation was a key theoretical mediator of intervention effects. Self-determination theory is an accepted theoretical underpinning of motivational interviewing (28) (see Table 1). Once these decisions were made by the intervention working group, in step 4 we operationalized goals, strategies, and needs into plans (e.g., step-by-step intervention manual for the counseling session, specific TMs and QQs, lottery prize structure). Guided by past work by Linnemayr and Rice (49), we conceptualized incentives as nudges rather than as motivators in and of themselves, attended to how nudges would align with participants' own health goals and motivation, and crafted delivery and communications for participants based on past research (e.g., highlighting the importance of participants' own HIV antiretroviral therapy decisions, using the prize wheel to add interest and excitement to the intervention). It was decided that all participants would receive at least some level of a lottery prize, whether they engaged in the TMQQ component at all and/or achieved HIV viral suppression or not (with larger prizes for those who engaged in the TMQQ component and who achieved HIV viral suppression, consistent with the behavioral economics approach). The intervention components were designed to be fair and transparent and also flexible and individualized (e.g., participants who required more than 16 weeks before checking their HIV viral load levels could delay spinning the prize wheel, but would not receive extra points in that time period). Behavioral economics recommends that financial incentives be provided as soon as possible after the behavioral objective is realized (50). Thus, one major issue addressed in the intervention mapping process was the challenge of applying a behavioral economics approach to a behavior that can take several months to achieve (HIV viral suppression). The length of the TMQQ period (16 weeks) was based on the typical length of time needed to achieve HIV viral suppression when PWH increase the frequency of HIV antiretroviral therapy doses or re-initiate HIV antiretroviral therapy with high levels of adherence (51). TMQQs were designed to be culturally and structurally salient in that they reflected or were consistent with the main “upstream” and cultural barriers African American/Black and Latino PWH experience to HIV viral suppression, as well as individual-level barriers, such as low health literacy, fear of side effects, and the high rates of stopping and starting HIV medication. TMQQs were intended to engage participants in the study over the period of time it takes to achieve HIV viral suppression, and to “nudge” participants toward the outcome, and as such were not designed to be overly complex or challenging to understand.

**Table 1:** Description of the components that make up the SCAP intervention.

**Component**	**Structure**	**Activities/objectives**	**Theoretical targets**
Intervention as a whole	–	- Provide content with high acceptability and feasibility - Provide an intervention distinct from existing programs to add interest and excitement - Foster HIV-related goal formation - Foster medication habit formation - Circumvent cognitive biases and heuristics - Motivate HIV viral suppression	Engagement, motivation (conscious and unconscious), habit formation, circumvent cognitive biases
Counseling session	<60 min, delivered by an interventionist to participants individually; guided by a manual	- Orient participants to the study - Communicate study ethos to foster engagement - Elicit participants' own HIV-related health goals and barriers to/facilitators of goals - Discuss habit formation - Introduce the TMQQ component and prize structure	Motivation (conscious) for HIV viral suppression
TMQQ	16 weeks in duration; an informational text message (TM) on HIV was sent once a week followed by a true/false quiz question (QQ) the next day	- Support engagement in the study over time - “Nudge” participants toward their HIV health goals, if any - Add interest and novelty to the intervention process - Participants earned 10 points for correct and 5 points for incorrect responses to the QQ (max. points 160)	Engagement; circumvent cognitive biases and heuristics; foster habit formation
Check-in contact	<30 min; conducted 8-weeks post enrollment	- Reinforce the main messages of the intervention - Demonstrate the prize wheel in a concrete fashion (with a practice spin and compensation up to $50) - Resolve any barriers to receiving TMs or responding to the QQs	Motivation; engagement
Lottery prize	Prize between $5 and $275 was allocated	- A prize wheel (similar to a roulette wheel) was spun to determine the prize amount - Prizes depended on (1) points earned in the TMQQ component, (2) whether HIV viral suppression was achieved, and (3) chance - Prize was intended to add interest and excitement to the process of achieving HIV-related goals - All participants received some level of prize	Motivation (unconscious); circumvent cognitive biases and heuristics

In step 5, the intervention components, described in more detail below, were field tested with the community advisory board (e.g., community board members on the intervention working group received the TMQQs and engaged in a “walk through” to reflect on their content and timing and suggest improvements, guidance was obtained on the level and structure of prizes). Based on the field test, intervention components were refined prior to implementation in this pilot study.

### Eligibility criteria

Inclusion criteria were: 1. age 18–65 years, 2. diagnosed with HIV, 3. resides in the New York City or Newark, NJ metropolitan areas, 4. can conduct research activities in English, 5. a recent laboratory report (from the past 2 months) indicates non-suppressed HIV viral load (> 200 pp/mL), 6. has a phone and can receive TMs, 7. has not participated in a local conditional economic incentive program for HIV viral suppression in the past month, and 8. had not participated in another study with our research lab in the past 6 months [At the outset of the study we defined non-suppressed HIV viral load as detectable viral load (≥20 pp/mL) but later changed that inclusion criterion to >200 pp/mL to align with the larger research literature (52). A total of 7.5% of participants in the present study had HIV viral load levels >20 but <200 pp/mL at baseline because they were enrolled with the original inclusion criterion]. In keeping with the present study as a pilot study, we did not include monolingual Spanish-speaking participants. Although race/ethnicity were not eligibility criteria, it was anticipated that >90% of participants would be Black or Latino given trends in past studies and the demographic characteristics of PWH in New York City and Newark [>75% Black or Latino (53, 54)]. Participation in studies outside our research lab was not assessed and was not an eligibility criterion; it was assumed some participants were engaging in other studies.

### Recruitment

Participants were recruited using a hybrid method that included direct recruitment by staff in community-based organizations serving PWH, advertisements placed in the medical research section of a local free newspaper, a recruitment registry comprised of individuals who had screened for or participated in past research studies at our institution, and peer-to-peer recruitment, where participants were compensated $15 for referring their peers to the study. Most participants (67.5%) were recruited from the recruitment registry (27/40), 20% (8/40) were recruited through peer referral, and 12.5% (5/40) from newspaper advertisements.

### Procedures and study design

#### Screening for eligibility and enrollment

##### First screening interview

Screening for study eligibility took place in two stages. For the first screening interview, potential participants contacted the study directly by phone. We obtained verbal informed consent following an IRB-approved script, and then participants engaged in a brief structured assessment in the computer-assisted personal interview (CAPI) format on the REDCap platform that assessed eligibility criteria. Sex assigned at birth and race/ethnicity were assessed but were not eligibility criteria.

##### Second screening interview

Those found preliminarily eligible at this stage were told they might be eligible for the research study, pending confirmation of non-suppressed HIV viral load on a recent laboratory report (HIV viral load assessed in the past 2 months). If potential participants were interested, research staff discussed strategies participants could use to obtain new or existing laboratory reports without cost. Prior to the suspension of in-person research activities due to COVID-19, participants brought a copy of the laboratory report to the field site. After in-person activities with human subjects were suspended, participants were asked to provide the laboratory report in an electronic format prior to the second screening interview or have their health care facility fax the report to the study. Faxes were received by a computer-based application on a password-protected computer. Challenges to obtaining laboratory reports included participants not recently attending HIV care visits and therefore not having a recent lab report, inconsistent access to cell phones or cell phone service being cut off, not being certain how to request a lab report from the provider, or not knowing how to send results to the study electronically. These barriers were overcome by walking participants through the process of obtaining records, helping them take a problem-solving approach to barriers, and offering to contact the provider directly (with participants' signed consent). Participants commonly required assistance obtaining the laboratory report (~75% of the time).

During the second screening interview, HIV viral load values were entered into REDCap and we then determined study eligibility based on HIV viral load values. Lab reports were scanned as needed and the electronic version was loaded into REDCap. No paper copies were retained, nor were electronic copies of records stored on computer hard drives, to protect participant confidentiality. Because participants requested their own records from providers or provided their records to the study, they were not required by the study to sign a Health Insurance Portability and Accountability Act (HIPAA) consent form. However, participants did sign a HIPAA form in cases where we were asked to contact the providers directly (we contacted providers in ~10% of cases). Participants received $15 for providing the laboratory report to the study and $10 for the second screening interview. For in-person visits we provided funds for round-trip local transportation in the form of a magnetic stripe card called a Metrocard.

##### Enrollment and baseline assessment

Those found eligible provided signed informed consent (if enrolled in person) or verbal informed consent if enrolled virtually due to COVID-19 restrictions and completed a structured baseline assessment battery in the CAPI format on the REDCap platform lasting ~60 min. Participants received $25 for the baseline assessment.

#### Intervention components that comprise the SCAP intervention

##### Counseling session

The intervention began with a counseling session grounded in the motivational interviewing approach that lasted ~60 min or less and was carried out by a trained clinical interventionist. The goals of the counseling session were to engage participants in the study, communicate the study ethos (no pressure and no judgment about their HIV antiretroviral therapy decisions), and to elicit participants' own HIV-related health goals, if any, including regarding HIV viral suppression, to thereby begin to foster durable intrinsic motivation for behavior change, which might align with the other study components (the “nudges”). The session also introduced participants to the concept of habit formation and to the procedures in TMQQ intervention component. Participants who did not wish to take HIV antiretroviral therapy and/or achieve HIV viral suppression at this time were encouraged to remain in the study. Participants were provided with a handout that explained the TMQQ component and the lottery prize structure. Participants received $25 for the counseling session. The TMQQ component began1week after the counseling session was completed.

##### TMQQ component

The TMs were intended to be relatively simple informational messages that would be relevant and interesting to PWH (e.g., “A lot of people in our SCAP community are worried about HIV antiretroviral therapy side effects. Side effects are real, but today's HIV medications are easier to take than ever”). Participants could receive TMs related to HIV or those related to health generally that did not mention HIV, depending on their preferences. Further, they could elect to have their name appear in the TMs.

Participants were trained in the TMQQ component at the end of the counseling session. Participants were guided in entering the SCAP phone number into their phone contacts, and a sample TM was sent to the participant, followed by a QQ which the participant was instructed to answer. Then, each week, participants first received an informational TM. Two days later, participants received a QQ that was based on the informational message. TMs generally included a link to a website or article where participants could get more information about the topic (10/16 messages included a link). The QQ was in the format of a true/false question [e.g., “Today's HIV medications have fewer side effects than HIV medications in the past. Press 1 for true, Press 2 for false. Respond to the text to earn points and prizes.” (answer: true)]. TMQQs were sent automatically by the Telerivet program, which also tracked response rates and the proportion of correct vs. incorrect responses. After answering the QQ, participants received an automatic TM informing them whether their response was correct or incorrect, and if incorrect, a link to the correct information was provided. Participants earned 10 points for correct responses to QQs and 5 points for incorrect responses. Thus, the maximum number of possible points over 16 weeks was 160. Participants who had not achieved HIV viral suppression by the sixteenth week and who wished to extend the TMQQ period could do so, but did not earn further points after the sixteenth week (~15% of participants wished to extend the TMQQ period). In short, responding to QQs generated points, and number of points earned increased the chances of winning a large prize for achieving HIV viral suppression at the first follow-up assessment. The TMQQs are provided in Appendix A.

##### Lottery prizes

Lottery prizes were received at the end of the 16-week TMQQ period. They ranged from $5 to $275 depending on (1) number of points earned in the TMQQ component, (2) whether HIV viral suppression was achieved, and (3) chance. Points were categorized into high (120–160 points), medium (60–119 points), or low levels (0–59 points). Participants at each point level had a 1/10 chance of winning the large prize and a 9/10 chance of winning the smaller prize.

As shown in Table 2, compensation levels for those who achieved HIV viral suppression were as follows: Those who earned the high level of points had a 9/10 chance of winning $75 and a 1/10 chance of winning $275. Those with points at the medium level had a 9/10 chance of winning $50 and a 1/10 chance of winning $175. Those with points at the low level had a 9/10 chance of winning $30 and a 1/10 chance of winning $150. Compensation levels for those who did not achieve HIV viral suppression were as follows: Those who earned the high level of points had a 9/10 chance of winning $15 and a 1/10 chance of winning $50. Those with points at the medium level had a 9/10 chance of winning $10 and a 1/10 chance of winning $40. Those with points at the low level had a 9/10 chance of winning $5 and a 1/10 chance of winning $30. Thus, all participants received a prize at the first follow-up interview, regardless of HIV viral suppression or level of engagement in the TMQQ component.

**Table 2:** Lottery prize compensation based on points earned in TMQQ component, viral suppression, and chance.

**Point level**	**Low probability—big prize**	**High probability—smaller prize**
**For those who achieved HIV viral suppression at follow-up**		
HIGH (120–160 points)	1/10 chance big prize ($275)	9/10 chance smaller prize ($75)
MEDIUM (60–119 points)	1/10 chance big prize ($175)	9/10 chance smaller prize ($50)
LOW POINTS (0–59 points)	1/10 chance big prize ($150)	9/10 chance smaller prize ($30)
**For those who did not achieve HIV viral suppression at follow-up**		
HIGH (120–160 points)	1/10 chance big prize ($50)	9/10 chance smaller prize ($15)
MEDIUM (60–119 points)	1/10 chance big prize ($40)	9/10 chance smaller prize ($10)
LOW POINTS (0–59 points)	1/10 chance big prize ($30)	9/10 chance smaller prize ($5)

#### Check-in contact and qualitative interview

At ~8-weeks post-baseline, participants engaged in a check-in contact (<60 min). Consistent with the present study as an exploratory effort, the check-in contact was a hybrid encounter that was intended first to reinforce the main messages in the intervention, demonstrate the prize wheel in a concrete fashion (with a practice spin), and resolve any barriers to receiving TMs or responding to the QQs. The goal of the practice spin and prize (up to $50, depending on points earned in the TMQQ component to date, but not based on HIV viral suppression) was to demonstrate the prize wheel process and reinforce the concept that responding to QQs allows participants to earn points that translate into higher probabilities of prizes [Those earning a high level of points at this mid-way period (60–80 points) had a 9/10 chance of winning $15 and a 1/10 chance of winning $50. Those with points at the medium level (30–59 points) had a 9/10 chance of winning $10 and a 1/10 chance of winning $40. Those with points at the low level (0–29 points) had a 9/10 chance of winning $5 and a 1/10 chance of winning $30].

Second, we elicited feedback on the acceptability and utility of the SCAP intervention in the format of a semi-structured in-depth qualitative interview. After the check-in activities were completed, participants engaged in a brief (20–30 min) in-depth semi-structured qualitative interview on their experiences in the project to date. These interviews were audio-recorded and professionally transcribed verbatim for analysis. Participants received $20 for the check-in contact along with the mid-way lottery prize, the amount of which was determined by the spin of the prize wheel.

#### Follow-up assessments and lottery prize

##### First follow-up assessment

Prior to the first follow-up assessment, participants were contacted and asked to provide a recent lab report with HIV viral load levels (regardless of whether they were HIV virally suppressed or not). When laboratory reports were obtained, the follow-up assessment was scheduled and carried out in CAPI in the REDCap platform (lasting ~30 min). Participants received $15 for providing the laboratory report and $25 for the follow-up assessment.

##### Determination of lottery prize amount

Participants then spun the prize wheel (if in person) or had it spun for them (if virtual) and earned the lottery prize based on points received in TMQQ component, whether they achieved HIV viral suppression, and chance.

##### Second follow-up assessment

Procedures for the second follow-up assessment were similar to the first: participants were contacted in advance to obtain the laboratory report, and then the follow-up assessment was scheduled and carried out. Participants' challenges obtaining laboratory reports increased during COVID, and compensation for providing the laboratory report was increased from $15 to $35. This change was approved by the IRB prior to implementation.

#### Quantitative measures

##### Sociodemographic and background characteristics

Structured instruments developed specifically for HIV-affected populations in high-risk contexts such as the population under study here were used to assess relevant quantitative domains, including age, sex assigned at birth, gender identity, sexual minority status (identifies as gay, lesbian, bisexual, queer, or other non-heterosexual), race/ethnicity, education level (high school graduate or equivalent or higher), history of homelessness (homeless over the lifetime, homeless in the past year), whether currently stably housed (that is, the residence is not temporary [such as a single-room occupancy hotel] or a location unfit for human habitation, including living on the streets), monthly household income < $1,000, whether covered by public insurance or health plan, and whether currently employed full- or part-time (55). We assessed a range of HIV indices using a version of the HIV Cost and Services Utilization Study instrument (HCSUS) (56) including: Years since first HIV diagnosis, whether perinatally infected with HIV, whether has taken HIV antiretroviral therapy in the past, years since first initiated HIV antiretroviral therapy, number times has stopped and started HIV antiretroviral therapy (a numerical response), the longest duration of sustained HIV antiretroviral therapy use in months (a numerical response), adherence to HIV antiretroviral therapy doses over the past month on a visual analog scale (VAS; range 0–100% of prescribed doses taken), if not on HIV antiretroviral therapy at enrollment, number of months since last dose (a numerical response). Patterns of substance use were assessed using the World Health Organization Alcohol, Smoking and Substance Involvement Screening Test (WHO ASSIST) which provides scoring algorithms to distinguish substance use at moderate-to-high risk vs. low-risk levels (57). We assessed engagement in any substance use treatment in the past (e.g., outpatient drug treatment, detox, inpatient drug treatment, methadone maintenance treatment program, 12 step or self-help meetings like AA or NA), an indicator of past concerns about substance use (recoded as yes if any substance use treatment was reported). Physical and Mental health were assessed using the SF-12 measure, a self-reported outcome measure assessing the impact of health on an individual's everyday life (58). We created T scores from the SF-12 items; namely, weighted linear composite scores using weights presented by Ware et al. (58). The normative mean for composite scores in the 1995 general U.S. population was 50. In addition to physical and mental health composite scores, we also used the SF-12 items to create the SF-6D preference-based measure of health described by Brazier and Roberts (59). SF-6D scores can range from 0.35 to 1.0 with higher values indicating better health. The average SF-6D score for an adult UK population in 1998 was 0.8.

##### Motivation

We assessed motivation for (1) HIV care attendance and motivation to (2) take HIV antiretroviral therapy (if taking HIV antiretroviral therapy at all at the time of enrollment) or increase HIV antiretroviral therapy adherence (if taking HIV antiretroviral therapy but not at a sufficient level to achieve viral suppression). Based on past research, motivation was conceptualized as how important a behavior or outcome is to an individual and how confident they are they can engage in the behavior or achieve the outcome (60). Importance of the behavior was rated on a 1–10 scale (e.g., On a scale of 1–10, how important is it to you today to significantly increase how often you take HIV medication, where 1 is not important at all, and 10 is extremely important?), followed by the participant's confidence that they could engage in the behavior (e.g., On a scale of 1–10, how confident are you that you could significantly increase how often you take HIV medication, where 1 is not at all confident and 10 is extremely confident?). Thus, “motivation” for a behavior was operationalized as the mean of the importance score and the mean of the confidence score and ranged from 1 to 10; higher values indicated higher motivation for the behavior (60).

##### Acceptability and feasibility

A version of the Client Satisfaction Survey (61) was adapted to the present study by the research team and reviewed by the community advisory board for comprehensiveness and clarity. The revised Client Satisfaction Survey was used to assess the acceptability of the study overall and of aspects of the intervention components that comprised the SCAP intervention. A total of 15 items were assessed such as “the SCAP staff understand the treatment needs of people of my racial, ethnic, or cultural group,” and “the chance to win a prize as part of the SCAP study played a role in my recent HIV medication decisions.” Items were rated on two types of Likert-scales depending on the item (poor, fair, good, very good, excellent, or rarely or never, sometimes, most times, and all of the time) and coded to reflect the proportion who endorsed the item as “very good to excellent” or “most times to all of the time.” An item was considered acceptable if 70% or more of participants endorsed it as “very good to excellent” or “most times to all of the time.” Some questions were asked at the second follow-up assessment only. Study feasibility was defined as proportion of participants attending assigned components. The study or a component was considered feasible if 70% of more of participants engaged in the activity.

##### Primary outcome

HIV viral load level and HIV viral suppression (≤200 copies/mL) were assessed by laboratory report from the participant's HIV primary care site.

### Qualitative guide

The check-in contact was carried out with participants following a semi-structured guide developed by the research team, which included experts on African American/Black and Latino PWH, behavioral economics, and the HIV care continuum. Structured as a series of suggested questions and prompts, the guide directed the interviewer from general to more specific questions in each of the following sections: (1) general overview of participant's experience in the project and any progress made with respect to HIV medication or otherwise (e.g., *How are you doing since I saw you last?, Are you* taking HIV medication or do you have plans to take medication?); (2) experiences with the TMQQ component (e.g., *What do you think about the text messages you have received? Are they easy to read? Hard to read? Too long? Too short?, Are they helpful in any way? If so, how? Are they unhelpful in any way?);* (3) experiences with the point system (e.g., *What do you think about the points system? Are the points helpful in any way? If so, how? Are they unhelpful in any way?);* (4) perspectives on habits and sustained HIV viral suppression *(e.g., The idea of SCAP is that you will develop health habits that will continue after SCAP is finished. Sometime people achieve HIV viral suppression and then stop taking HIV medications. Why do you think that is?);* (5) perspectives on the final prize *(e.g., Are the main/final prizes as they are structured [taking into consideration points and viral suppression, with a chance element] motivating? Interesting? Confusing?*). Then, the practice spin was carried out and participants' plans for the subsequent 8 weeks of the TMQQ component were reviewed.

#### Quantitative data analysis

Descriptive statistics were presented by time of assessment (baseline, follow-up 1, and follow-up 2), with percentages for categorical variables and means and standard deviations for continuous variables. Following recommendations from NIH (62) and in the methods literature (63), we did not perform null-hypothesis significance testing with these pilot data. All analyses were conducted with the R statistical computing environment (64).

#### Qualitative data analyses

Analyses of qualitative data followed a directed content analysis approach that was both inductive and theory-driven (65). We started with an initial list of “start codes” and their operational definitions that was generated by the primary qualitative analyst, who is a medical anthropologist. This initial start code list was informed by the theories and perspectives framing the study. Codes were generated that reflected structural barriers (e.g., quality of housing, poverty), culture and race/ethnicity (e.g., experiences of discrimination, medical distrust, counter-narratives); substance use management; autonomy, competence, and relatedness; and other factors known to promote or impede engagement along the HIV care continuum (e.g., mental health distress). Using this scheme, the primary analyst coded interview transcripts along with an additional trained qualitative researcher. During the coding process, codes were refined, clarified, and/or broadened; for example, when new codes were identified. Discrepancies in codes and coding between the data analysts were resolved by consensus. Then, the interview transcripts were recoded using the final coding frame. Further, a subset of transcripts were coded using the final coding frame by three other members of the research team. Codes were then combined into larger themes and sub-themes in an iterative process led by the two main data analysts and in collaboration with an interpretive community of research team members, which included cisgender men and women, people who identify as transgender, gender non-binary, or gender-fluid, people from White, African American/Black, Asian, and Latino backgrounds, and PWH (66, 67). Methodological rigor of the analysis was monitored continually in several ways. An audit trail of process and analytic memos was maintained (68). Analysts engaged in debriefing sessions approximately monthly with the interpretive community. The primary analysts and the interpretive community attended to the potential effects of the team's positionality related to power and privilege, sex, gender, race/ethnicity, health, and socioeconomic status throughout the data collection process through reflection and training that focused on how these factors might affect interviewing and data analytic processes (48, 69).

### Data integration procedures

Data integration followed procedures outlined by Fetters et al. and used the joint display method (70). A joint display is a state-of-the-art visual tool (i.e., a side-by-side visual presentation of results) to integrate data sources. The process brings about new insights beyond the information gained from the separate quantitative and qualitative results. Data integration is carried out by an interpretive community made up of members of the research team in an iterative process in which each joint display table reveals insights about the merged findings that shape subsequent iterations. Thus, joint displays are both a method and a cognitive framework for data integration and facilitate the production of new inferences (70). Beginning with the major quantitative findings, the interpretive community assessed areas of convergence and divergence between the quantitative results and the primary themes in the qualitative data. To do so, we used an informational matrix to compare results at a granular level (finding by finding) (70). Then, we explored primary qualitative findings that may not be present in the quantitative results. The results from this data integration effort were summarized and presented in a joint display table.

## Results

### Sociodemographic characteristics of the sample

Table 3 shows demographic and other characteristics of the sample at baseline. We describe select characteristics here. Participants were 50 years old, on average (SD = 10.8 years). Approximately half (58%) were assigned male sex at birth, and two-thirds (68%) were African American or Black. Most (78%) had achieved a high school diploma or higher. Almost all (90%) had been homeless over their lifetimes, but 93% were stably housed at the present time. Indications of poverty included household income < $1,000 a month (70%), eligibility for safety-net health insurance (97.5), and only 10% were employed. Participants had been diagnosed with HIV 22 years ago on average (SD = 8 years). All had taken HIV antiretroviral therapy in the past, and almost three-fourths (73%) were taking HIV antiretroviral therapy at enrollment, although not at a level sufficient to achieve HIV viral suppression. Participants had long histories of stopping and starting HIV antiretroviral therapy: they first initiated HIV antiretroviral therapy 20 years previously, on average (SD = 7 years), and had stopped and re-started medication 19 times, on average (SD = 48 times). Substance use at moderate-to-high risk levels was found among 35% for alcohol, 40% for cannabis, and 45% for cocaine.

**Table 3:** Sociodemographic and background characteristics and HIV-related health factors (*N* = 40).

	**Mean (SD) or %**
Age in years (M, SD)	50.1 (10.8)
Age range [min, max], in years	25.0, 62.0
*Sex, sexual orientation, and gender identity*	
Male sex assigned at birth	57.5%
Female sex assigned at birth	42.5%
Sexual minority (bisexual, homosexual, queer, gay, lesbian)	37.5%
Transgender, gender fluid, gender identity	0%
African American/Black (non-Latino/Hispanic)	67.5%
Latino/Hispanic	25.0%
High school graduate/equivalent or higher	77.5%
Homeless over the lifetime	90.0%
Homeless in the past year	20.0%
Currently stably housed	92.5%
Monthly household income < $1000	70.0%
Covered by public “safety net” insurance or health plan	97.5%
Currently employed full- or part-time	10.0%
*HIV history and HIV health status indicators*	
Years living with HIV/years since HIV Diagnosis (M, SD)	22.2 (7.48)
Range of years living with HIV [min, max]	3.00, 31.0
Perinatally infected with HIV	12.5%
Has taken HIV antiretroviral therapy in the past	100%
Years since first initiated HIV antiretroviral therapy (M, SD)	19.7 (7.32)
Range of years since initiated HIV antiretroviral therapy [min, max]	3.00, 31.0
Number of HIV antiretroviral therapy starts (range 0–288 times) (M, SD)	18.7 (48.2)
Longest duration of sustained HIV antiretroviral therapy, in months (range 2–204 months) (M, SD)	43.6 (53.0)
Adherence to HIV antiretroviral therapy in past month (range 0–100% of doses) (M, SD)	54.4 (38.3)
Taking HIV antiretroviral therapy at enrollment	72.5%
If not on HIV antiretroviral therapy at enrollment, number of months since last dose (M, SD)	7.15 (3.76)
Satisfaction with HIV care (range 0–100) (M, SD)	80.75 (19.95)
*Substance use patterns (WHO ASSIST)*	
Alcohol use at a moderate-to-high risk level	35.0%
Cannabis use at a moderate-to-high risk level	40.0%
Cocaine use at a moderate-to-high risk level	45.0%
Polysubstance use (2+ substances excluding tobacco and alcohol) at a moderate-to-high risk level	0.0%
Any substance use treatment over the lifetime	75.0%

### Enrollment and feasibility

Table 4 provides data on study screening, enrollment, and participation in study activities (feasibility). We screened 137 individuals between December 2, 2019 and August 5, 2020, enrolling 40 participants in the study. Of the 137 individuals who began the first stage of screening, 126 (92%) were eligible at that stage, but only 43 (34% of those eligible in the first stage) initiated the second and final stage of the screening process. The main reasons for participants failing to complete the second screening interview after being found eligible in the first screening interview were declining to provide a laboratory report (44.6%), being unable to provide a lab report (41.0%), or being lost to follow-up (14.5%). Most of those who initiated the second stage of screening were enrolled (*N* = 40/43; 93%). Most (97.5%) completed the check-in contact. At that time, most (87.2%) had earned the highest level of points on the TMQQ component (between 60 and 80 points). Of those with the highest level of points (*N* = 34), 7 won the large prize and 27 won the small prize. Almost all participants (97.5%) answered at least one QQ. Participants answered 13 of 16 QQ on average (mean = 13.3; SD = 3.9) and earned an average of 127 out of 160 possible total points (SD = 38 points). Most of the quiz questions were answered correctly (mean = 11.8; SD = 3.7). Most (N = 38, 95%) completed the first follow-up and most (N = 32, 80%) also completed the second follow-up assessment. The average time average time dedicated to the counseling session was 40 min (range 20–60 min), with no difference between racial/ethnic groups.

**Table 4:** Enrollment and feasibility.

	***N* (%) or M (SD)**
**Screened for eligibility (pre-screening)**	137
Ineligible	11/137 (8.0)
Eligible for Screen 2	126/137 (92)
**Did not conduct second screening interview**	83/126 (65.9)
Reasons for not conducting second screening interview	
Declined to provide lab report	37/83 (44.6)
Unable to provide lab report	34/83 (41.0)
Lost to follow-up	12/83 (14.5)
**Conducted second screening interview**	43/126 (34.1)
**Eligible for the study after screen 2**	40/43 (93.0)
Ineligible due to suppressed viral load	3/43 (7.0)
**Enrolled/received baseline**	40/40 (100)
**Completed intervention session 1**	40/40 (100)
**Check-in contact completed**	39/40 (97.5)
High points (60–80 points)	34/39 (87.2)
Won large prize ($50)	7/34 (20.6%)
**Text messages (TM) and quiz questions (QQ)**	
Answered at least one QQ	39/40 (97.5)
Number answered [Mean, SD]	13.3 (3.9)
Number correct [Mean, SD]	11.8 (3.7)
Final points, max. = 160 [Mean, SD]	127 (38.0)
**Follow-up assessment 1 (FU1)**	
Follow-up 1 assessment completed	38/40 (95.0)
Follow-up 1 lab report received	35/40 (87.5)
**If achieved HIV viral suppression at FU1**	**16/35 (of lab reports received)**
High points (120–160 points)	16
Won large prize ($275)	2
Medium points (60–119 points)	0
Won large prize ($175)	0
Low points (0–59 points)	0
Won large prize ($150)	0
**If did not achieve HIV viral suppression at FU1**	**19/35 lab reports received**
High points (120–160 points)	12
Won large prize ($50)	4
Medium points (60–119 points)	5
Won large prize ($40)	2
Low points (0–59 points)	2
Won large prize ($30)	0
**Requested to delay prize after FU1**	16
Months delayed	2
Suppressed viral load when spun for prize	6/16 (37.5%)
**Follow-up assessment 2 (FU2)**	
Follow-up 2 assessment completed	32/40 (80.0%)
Follow-up 2 lab report received	27/40 (67.5%)

### Acceptability and self-reported influence of SCAP on behavior

Table 5 shows responses to items assessing acceptability at the second follow-up assessment. We review some of the findings here. About three-quarters rated services received in the study as very good or excellent (75%). Almost all (97%) reported the study understands the treatment needs of people of their racial, ethnic, or cultural group most times to all of the time. Approximately two-thirds (69%) of participants said the chance to win a prize played a role in their recent HIV antiretroviral therapy decisions somewhat to a great deal, and 72% said that they tried to achieve HIV undetectable viral load in order to win a prize (somewhat to a great deal). Moreover, 75% reported receiving TMQQs to earn points played a role in their recent HIV antiretroviral therapy decisions (somewhat to a great deal). A total of 75% reported that the counseling session played a role in their recent HIV antiretroviral therapy decisions (somewhat to a great deal). Overall, 75% of participants reported the SCAP study played a role in their recent HIV antiretroviral therapy decisions (somewhat to a great deal).

**Table 5:** Intervention acceptability at the final follow-up assessment (*N* = 32).

	***N* (%)**
Overall, I think the services in the SCAP study are very good to excellent	24 (75.1%)
Overall, I think the text messages I received as part of the SCAP study are very good to excellent	23 (71.9%)
The SCAP staff respect my privacy most times to all of the time	31 (96.9%)
The SCAP staff understand the treatment needs of people of my racial, ethnic, or cultural group most times to all of the time	31 (96.9%)
(If female) The SCAP staff understand the needs of women most times to all of the time	14 (87.6%)
The chance to win a prize as part of the SCAP study played a role in my recent HIV medication decisions somewhat to a great deal	22 (68.8%)
Because of the chance to win a prize as part of the SCAP study, I tried to achieve HIV undetectable viral load somewhat to a great deal	23 (71.9%)
Receiving text messages and answering quiz questions to earn points as part of the SCAP study played a role in my recent HIV medication decisions somewhat to a great deal	24 (75.1%)
Because of the TMQQs, I took HIV medication more often than I did in the past somewhat to a great deal	22 (68.7%)
Because of the TMQQs, I tried to achieve HIV undetectable viral load somewhat to a great deal	25 (78.1%)
Meeting with the SCAP staff to discuss my goals and learn about habits as part of the SCAP study (the counseling session) played a role in my recent HIV medication decisions somewhat to a great deal	24 (75.0%)
Because of meeting with the SCAP staff to discuss my goals and habits (the counseling session), I took HIV medication more often than I did in the past somewhat to a great deal	21 (65.7%)
Because of the meetings with SCAP staff (the counseling session), I tried to achieve HIV undetectable viral load somewhat to a great deal	23 (71.9%)
Overall, the SCAP study played a role in my recent HIV medication decisions somewhat to a great deal	24 (75.0%)

### Motivation

Although the study was not powered for efficacy, we examined trends in motivation and key outcomes. Ratings of motivation for both taking HIV antiretroviral therapy and attending HIV care increased from baseline to follow-up (Table 6).

**Table 6:** Motivation, health-related quality of life, HIV care engagement, and HIV viral load over time [mean (SD) or percent].

	**Baseline** **(*n* = 40)**	**First follow-up** **(*n* = 38)**	**Second follow-up** **(*n* = 32)**
**Motivation (0–100)**			
Motivation for HIV Care	88.5 (13.5)	97.1 (6.6)	93.2 (8.8)
Motivation for High HIV Medication Adherence	73.1 (26.6)	92.2 (13.7)	92.0 (11.8)
**SF-12 health-related quality of life**			
SF-6D Health Utility score	0.69 (0.21)	0.74 (0.21)	0.77 (0.17)
SF-12 Physical Health T-score	42.0 (12.7)	45.8 (11.3)	43.8 (10.4)
SF-12 Mental Health T-score	46.7 (11.6)	49.6 (10.6)	53.0 (9.6)
**HIV treatment engagement**			
Self-reported HIV medication adherence (0–100)	49.0 (39.8)	64.4 (40.0)	77.0 (30.7)
HIV medication taken in past 3–4 Weeks	73%	77%	91%
**HIV viral load**			
log_10_ HIV viral load^†^	3.57 (0.97)	2.89 (1.41)	2.62 (1.55)
Suppressed (viral load <200)	7.5%	45.7%	51.9%

† Viral load results obtained for 40, 35, and 27 participants at baseline, first follow-up, and second follow-up, respectively.

### Health-related quality of life

Health utility, physical health, and mental health scores on the SF-12 showed improvements from baseline to follow-up (Table 6).

### HIV treatment engagement and HIV antiretroviral therapy adherence

Both the percentage of participants taking any HIV antiretroviral therapy recently and self-reported adherence to HIV antiretroviral therapy increased from baseline to follow-up (Table 6).

### HIV viral load by laboratory report

HIV viral load decreased from baseline to follow-up and the percentage of participants with suppressed viral load increased from baseline to follow-up (Table 6). We also explored whether participants sustained HIV viral suppression among those who provided laboratory reports at follow-up *1 and* follow-up *2:* 4/25 (16%) were HIV virally suppressed at follow-up 1 but not follow-up 2 (did not sustain viral suppression), 10/25 (40%) were HIV virally suppressed at follow-up 1 and follow-up 2 (sustained viral suppression), 4/25 (16%) achieved HIV viral suppression at follow-up 2 but not follow-up 1 (achieved viral suppression late), and the remainder 7/25 (28%) did not achieve viral suppression (data not shown on Table 6).

### Qualitative results

We summarize participants' views on barriers to and facilitators of engagement along the HIV care continuum, including sustained HIV viral suppression, in the context of the COVID-19 pandemic in this section. We found primary barriers to engagement along the HIV care continuum included mental health issues, substance use, forgetting to take medication, HIV medication fatigue, fear of side effects, disliking pills, and missing HIV antiretroviral therapy doses due to not wanting to be reminded of one's HIV status. The COVID-19 pandemic had mixed effects on study participants. It presented new barriers to engagement in HIV care and HIV antiretroviral therapy adherence. Many participants noted difficulties related to COVID-19 such as clinic closures, bureaucratic obstacles such as disruptions in insurance coverage, and scheduling problems, and the inability to maintain consistent routines, including remembering when to take HIV antiretroviral therapy (i.e., at the same time each day). Many participants noted that financial insecurity and housing precarity dramatically increased as a result of COVID-19 and these proved to be two of the most prominent COVID-19-related barriers to HIV antiretroviral therapy adherence and HIV viral suppression. The COVID-19 pandemic also resulted in a number of new incentives to achieve optimal health, including HIV viral suppression, to prevent potential adverse effects of COVID-19. Participants noted that anxieties related to contracting COVID-19 created an impetus to maintain or even improve one's health. Citing concerns related to the perceived combined risks of HIV and COVID-19 infection, as well as a desire to reconnect with friends, family, and loved ones once COVID-19-related restrictions are relaxed, many participants noted an increased awareness of the risks associated with HIV antiretroviral therapy non-adherence. Although many of these barriers have been documented extensively in the literature, it is worth noting here that, for many participants, the COVID-19 pandemic has both presented new barriers to HIV antiretroviral therapy adherence as well as a number of newly emerged incentives to achieve optimal health, including HIV viral suppression.

Specific themes related to perspectives on aspects of the SCAP intervention and representative quotes are presented in Table 7 and summarized here. We found that the SCAP intervention was experienced as something new and different from past programs participants had engaged in, and overall, was experienced as relevant, motivating, and engaging. The chance to earn a prize was perceived as a motivator for joining the study, but not a reason to change HIV management behavior. The TMQQs were acceptable but could be improved in a number of respects. Last, the system to determine the prize amount was confusing and warrants simplification.

**Table 7:** Summary of qualitative results at check-in contact.

**Theme or finding**	**Representative quote(s)**
**Effects of the SCAP intervention on motivation to achieve HIV viral suppression:** Almost without exception, participants highly valued achieving and sustaining HIV viral suppression. Many noted the COVID-19 pandemic had increased their motivation to maintain good health, including becoming HIV virally suppressed. The SCAP intervention was experienced as something new and different from past programs they had engaged in.	The feeling, you know—I'm a much more healthier because, I mean, it was kind of touch and go when I got sick and everything. But that's when the coronavirus had hit, you know, and it was just really, really not a good place to be [not virally suppressed], at the time, you know?
**The chance to earn a prize was a motivator for joining the study, but not a reason to change HIV management behavior**. Some participants were adamant that in general there is not or at least should not be a direct link between financial incentives and motivation to adhere to ART, but that the possibility of winning the prize and receiving financial compensation was a major reason they participated in the project, particularly during the COVID-19 pandemic.	Well, it depends on the individual. It actually depends on why you're here. If you are here just for the money, that I can see why it's an issue, but if you are here for the benefit of the program and the money is just icing. You know, it's just a little incentive. This is coffee money for me, you know? I'm not looking to buy watches or phones off of the study money.
**The counseling session** was experienced as a conversation, and personal, individualized contact with staff was appreciated.	(The session was) just to kind of see where people are at, you know? It's kind of like a one-on-one therapy type of session.
**Engagement with TMQQs and overall utility:** Participants generally found the TMs informative and helpful overall. Some participants also reported actively engaging with the quizzes and/or information in their daily lives, and that the TMQQs prompted them to gather additional information about HIV management.	I think that's good, too, because then now I have to look it up, you know, so that puts me into research mode, and I'll look up stuff and then I'll find other stuff that I didn't know along the way.
**TM Frequency:** Participants responded favorably to receiving TMs, and noted being either satisfied with the quantity of messages, or even desiring to receive them more frequently. Some participants appreciated the brevity of the TM messages. Many participants reported having frequent difficulties with their cellular phone plans, most commonly attributed to exhausting their “minutes” and/or data plan allotment, and also the inability to make payments on time or at all.	Oh, I enjoy it [the TMQQs]. I think it should be more frequent like maybe Monday, Wednesday and Friday. Like have a question. And I think it's fun. I look forward to sometimes that text to answer questions, be reminded that there's other people out there that are like-minded and I hope the situation gets better. And it just makes me feel good, you know, that's all. [The TMQQs remind me there are] people who care about you, they're still thinking about you.
**TM Content and Comprehensibility:** Participants generally found TMs both “logical and informative,” and in some cases noted that receiving regular TMs served to remind them about taking HIV medication, and in some cases was a source of new information.	I think it's perfect just the way it's been, because it's not just focusing on one area. It's a multiple-choice of questions and situations, and it gets you to start thinking about some things and how you can apply this to your life, you know?
**TM difficulty:** A small number of participants expressed that, based on their preexisting knowledge about HIV, they found that TMQQs were not sufficiently challenging.	I've tried everything. I haven't missed nothing new, it sounds like—like what you told me about the injections that are coming [injectable ART]. That wasn't one of the questions. But like I said, somebody who hasn't [been well-informed], yeah, they might be quite helpful. But for somebody who has, like I have done all that, so no, it hasn't been. That's why I knew what the answers were! (laughs) […] I don't know. You are trying to be helpful, so like I said, for people who don't know, yes. For people who are more advanced, maybe you have to put some other things in there to try and change it up.
**There was room for improvement in TMQQs:** A small number of participants found some of the messages challenging or confusing. Since they wanted to earn their points, this caused stress. Yet, for most, even disagreeing with the wording of a question did not prevent them from actively engaging with the TMQQ.	What I like about them is that—very few of the questions are challenging to me. And that's only because I've done a lot of studying. I've been a peer educator. So a lot of those—it's funny how they're talking about—they just texted me. Hold on. I know I'm going to pass [get the QQ correct], anyway. It says: True or false question. Using a pill box will only make it harder to remember to take my medication? See, that's an opinionated question. That's a question based off an opinion. Because some people might feel that it's harder and some people might feel that it's easier. I don't like questions like this. “Only make it harder.” I don't know if it's going to make it harder. I can't speak for everybody. I'm going to look it up, though. I'm going to look it up. I want my 10 points.
**TMQQs were perceived as impersonal by some:** Some participants indicated the need for more open-ended, individually focused, and therapeutic questions, especially in the context of social isolation during COVID-19. Some participants offered suggestions regarding the relatively impersonal nature of some of the TMQQs. For these participants, TMQQs could be augmented with those that are more therapeutic and/or interpersonal. Specifically, participants expressed a desire for TMs that were more directly oriented to mental health issues, particularly as they pertain to the COVID-19 pandemic, and issues not directly related to HIV.	It could be more personal. […] Because this is just asking questions about [HIV], it could be more personal, I think personally [ask about] how do you feel and how do you live? I didn't see a person, I didn't see like a personal attachment. Just a true or false answer is not [personal contact]. […] And you want somebody to be interested in you—saying, yo, this is not just about a virus, because most people that's got the virus and go to programs hear about it all day. Are they knowledgeable of it? Not at all. They still beat around the bush. Some of them hear this and hear that and hear this. But do they know the facts? There's a difference. And then how the facts affect them.
**Financial Incentives were appreciated:** Participants overwhelmingly noted that the financial incentives provided by SCAP were not only appreciated, but were instrumental in being able to meet their daily needs, and reported finding the prize amounts to be appropriate or even generous, if the prize structure was not at least somewhat confusing (see below).	I think [the point system and financial incentives are] a good thing. It's a great motivational tool, you know? I think that it helps someone get more involved and more in tune to the overall [intervention] experience. And, I mean, it is nice getting a reward like this, basically considering, going back to the current situation that has affected so many areas of people's life [COVID-19], especially financially. You know, it's been a struggle.
**The point system/prize wheel was confusing:** Overall, participants' responses indicated a significant amount of confusion regarding the intervention's point system and related financial incentives, even when they nonetheless felt the messages served as a successful motivation tool in general.	I was confused about that. I mean I have the paper [an infographic describing the points and prizes]. It's somewhere in this house but I cannot get to them at this moment in time. However, I remember that if your count changes, if it goes up you get a certain amount of points or if you answer all the questions right you get a certain amount of points.
Some did to perceive the point system and allocation of prizes for all participants as fair. A modest number of participants questioned the overall fairness of the point system, suggesting that individuals who choose not to fully participate in the intervention should be rewarded less than those genuinely investing their time and energy. Most participants found the lottery prize interesting and exciting, but some suggested a fixed prize amount would be preferable. Since prize amounts were based on chance and points earned in TMQQs, this finding reflects the lack of clarity in the prize structure.	I'm going to be honest. I think it kind of sucks that I can be—if I'm getting 10, 10, 10, 10, then award me with what I'm actually winning. Why do I need to spin a wheel or something like that? Award me with—if I'm answering correctly, and I'm doing the right things, I would assume so. For those people who don't want to participate, why should they get rewarded at all? Because I try to make sure [to respond to the TMQQ]—like I said, my phone is acting up, and I try and make sure I always answer when you do text me, because I do want to participate in this. For me, like you said, for those who don't participate, I do not understand that [they would get any compensation]. They can get the question wrong. I understand that, too. That's fine. They still participated. But for those who don't answer at all, hey, that's not right for them still to get to spin.

### Integration of qualitative and quantitative results

The interpretative community compared the major quantitative findings to qualitative findings, working domain by domain, and created a joint display table by consensus (Table 8). Because the qualitative in-depth interviews were semi-structured and therefore allowed for exploration of emergent themes, we did not assume results from the qualitative effort would reflect every quantitative domain. We highlight a subset of the integrated findings presented in Table 8 in this section. Qualitative and quantitative results were generally congruent. Overall, qualitative data shed light on and extended quantitative results, and added richness and context to the quantitative results. For example, we found high intervention acceptability in quantitative results and qualitative analyses yielded complementary findings: we found participants appreciated that the intervention was new and different from past programs they had engaged in. They valued the chance to have “conversations” with staff (in the counseling session). The TMQQ component was engaging and interesting. In some cases, qualitative results suggested unexpected or unintended results including the intervention's mechanisms of action (e.g., participants understood that TMQQs were sent automatically but still commonly experienced them as a source of support and caring). The joint display analysis also produced ways the SCAP intervention could be improved (e.g., participants valued the chance to have conversations with staff and some suggested that more such opportunities [more counseling sessions] would be welcome). Taken together, the joint display analysis provided insights into how the intervention was received, participants' experiences with the content, and ways the intervention could be enhanced in future research.

**Table 8:** A joint display organized by the primary research questions and including emergent findings.

	**Quantitative findings assessed at the end of study**	**Participant experiences from qualitative research assessed mid-way through the study**
Acceptability—overall	>70% found the intervention very good to excellent	Participants appreciated that the intervention was new and different from past programs they had engaged in. They valued the chance to have “conversations” with staff. The TMQQ component was engaging and interesting.
Acceptability—Session	>70% reported meeting to discuss goals and learn about habits (the counseling session) influenced efforts to achieve HIV undetectable viral load somewhat to a great deal	Participants were socially isolated. The counseling session was very much appreciated and seen as a valuable and needed conversation with staff. Findings suggest that the counseling session and the approach taken in the intervention grounded in the integrated conceptual model played a role in participant engagement in the study and helped build a relationship with the project.
Acceptability—TMQQ	>70% found the TMQQ component very good to excellent	Participants understood that TMQQs were sent automatically but still commonly experienced them as a source of support and caring. Participants would have appreciated more personal interactions with the staff.
TMQQ—utility	–	TMQQs were generally found informative, thought-provoking, and useful. In a small number cases, participants did not necessarily agree with the TMQQ message (#12, #15). TMQQs served as a reminder to take one's HIV medication.
TMQQ—difficulty	–	Many participants noted the TMQQs were not sufficiently challenging. However, the information could still serve as a helpful reminder.
TMQQ—frequency	–	Participants were generally satisfied with the frequency of TMQQs but noted more frequent TMQQs would be welcome. Twice a week might be optimal.
TMQQ—other	–	Brevity of messages and true/false quiz format was generally acceptable.
Lottery prize	>70% reported the chance to win a prize influenced their efforts achieve HIV undetectable viral load somewhat to a great deal	The chance to earn a lottery prize was a motivator for enrolling in the study. The lottery prize was not a primary motivator for HIV medication use, but was appreciated. The chance to earn and win financial incentives was appreciated by all participants. The chance to spin the prize wheel was generally exciting.
		The chance to earn larger prizes based in part on chance was experienced as disappointing for some, and some participants suggested that all who responded to TMQQs were entitled to the large prize.
		The structure of earing points to increase the probability of earning a prize was unnecessarily confusing to some. Participants did not necessarily understand the structure in advance of receiving their prize, but generally satisfied with the prize they received. There is utility to providing all participants with some level of prize, regardless of points and viral suppression.
Mechanisms of action	Trends indicate higher levels of motivation for HIV care and medication from baseline to follow-up	Participants generally understood the importance of HIV viral suppression and believed suppression was a worthy goal. Findings suggest the “nudge” from intervention components was useful to many.
Evidence of efficacy	Trends indicate lower HIV viral load levels and higher rates of HIV viral suppression from baseline to follow-up	NA (Qualitative interviews were carried out prior to assessments of HIV viral load.)
Other findings	–	Participants valued the chance to have conversations with staff and some suggested that more such opportunities (more counseling sessions) would be welcome.
		Less disparity between high and low prize amounts, or smaller prize amounts, could be just as acceptable as the current prize structure.

## Discussion

Persistent racial/ethnic disparities in HIV morbidity and mortality signal the need for new approaches to foster consistent engagement along the HIV care continuum (11). Further, new emerging theories and technologies highlight the promise of efficient low-touch and technology-driven interventions for PWH (22–24). The present mixed-methods pilot study is exploratory and takes the first step in applying principles of behavioral economics as part of a behavioral intervention to address the problem of unsuppressed HIV viral load among African American/Black and Latino PWH recruited in community-settings. Thus, the present study addresses a significant gap in the literature, since most prior studies of conditional economic incentive interventions were conducted in clinical settings and/or included or primarily focused on PWH taking HIV antiretroviral therapy and with viral suppression (39, 40).

We found the SCAP intervention, comprised of a single motivational interviewing counseling session, 16 weeks of TMQQs, and a conditional economic incentive in the form of a lottery-type prize, showed high levels of acceptability and feasibility. This pilot study was not powered to detect efficacy, but we used the quantitative and qualitative results to understand participants' experiences with the intervention, what aspects appear promising, and where improvements or modification are needed. For example, we observed that rates of HIV viral suppression, the intervention's primary outcome, increased during the study period (although we did not examine statistical significance in this pilot study) and the qualitative results yield insights into the intervention's mechanisms of action and effects.

Overall, study findings suggest that in many cases the multi-component SCAP intervention was successful in motivating and/or “nudging” participants toward HIV viral suppression (52% achieved viral suppression at the second follow-up assessment), and that conditional economic incentives in the form of prizes commonly aligned with participants' own motivation for increasing antiretroviral therapy adherence to achieve HIV viral suppression. Moreover, findings suggest that a substantial proportion of participants (40%) sustained HIV viral suppression after the intervention ended and the lottery prize was received. While participants did not typically attribute the achievement of HIV viral suppression to the possibility of winning a financial prize, the prize did motivate initial engagement in the study, and added excitement and interest in the study, consistent with behavioral economic theory (49). TMQQs are feasible and appear to have been useful in keeping participants engaged in the study, and have other unanticipated effects as well, such as prompting some participants to learn more about HIV management, and serving as a reminder to take HIV antiretroviral therapy. We further interpret study findings in the context of the early days of the COVID-19 pandemic in New York City, a COVID-19 epicenter. We found the COVID-19 pandemic had mixed effects on participants. On the one hand, the pandemic created challenges to accessing HIV care due to clinic closures, medications, and ancillary services such as substance use treatment and increased financial insecurity and housing precarity in this population. On the other, participants noted that anxieties related to contracting COVID-19 created an impetus to maintain or even improve health.

### Lessons learned about the SCAP intervention

Results highlight aspects of the study methods and SCAP intervention that appear promising, as well as ways it can be improved. Study participants resided in high-risk contexts characterized by chronic poverty, which created challenges accessing medical records and maintaining consistent cell phone access. The SCAP intervention integrated motivational interviewing counseling with TMQQs and lottery prizes. Participants clearly valued the personal interactions with study staff, including in the counseling session, underscoring the view of the intervention working group that planned the intervention that a completely automated intervention may not be optimal for this subpopulation of PWH. The behavioral economic and motivational interviewing approaches appeared highly complementary since both approaches are designed to build motivation and support behavior change but do so in different ways. Participants suggested that additional such personal counseling opportunities would be welcome, along with interactive TM components (that is, two-way TM communications where participants communicate with a staff member in real-time) in addition to automated TMQQs. We found it is challenging to create TMQQs that are neither too easy nor too challenging for participants. The present study did not seek to make TMQQs challenging, as the purpose of the component was to maintain engagement over the 16-week period. Nonetheless, refinement of the TMQQ component is warranted. The lottery prize added interest and excitement to the study and was a primary reason participants joined the study. In other words, it facilitated the recruitment efforts, because the idea of winning prizes captured participants' attention.

### Limitations and implications for future research

The study has strengths, including the mixed methods approach and evaluation of the primary outcome by an objective measure, and also a number of limitations. In keeping with the exploratory nature of the study, the sample size is modest, and we did not include a control group, which limits inferences that can be drawn from the results. The sample did not include monolingual Spanish-speaking participants, which limits the generalizability of study findings to Latino PWH as a whole. An additional limitation is the possible influence of social desirability bias on findings. We sought to minimize social desirability bias during the qualitative interview process by asking general questions first and reminding participants they could and should feel free to decline to answer any question without penalty, and by using objective laboratory reports to assess the primary outcome. As noted in the Methods Section, participants had the option to extend the TMQQ period (without earning additional points) and delay spinning the prize wheel. Such variation from the protocol has the potential to reduce internal validity. The small sample size in the present study does not allow us to examine such effects but we will attend to them in future research. The present study does, however, highlight the utility of allowing deviations from the protocol in a structured and standardized fashion. Further, since we tested SCAP as a multicomponent or “packaged” intervention, the design does not allow us to determine which of the components contributed to intervention efficacy with precision. In future research we will examine the effects of individual components and their interactions in designs such as factorial experiments, grounded in the multiphase optimization strategy (71). The qualitative assessment was conducted mid-way through the study and participants' views on components including the lottery prize structure may have evolved after receiving the final prize. The sampling method and study procedures (such as the requirement for participants to provide their own laboratory report and to have their own phones to receive TMQQs), along with the emergence of the COVID-19 pandemic during the study may have introduced bias. Future studies can provide HIV viral load testing and cell phones to participants to eliminate these biases. Further, the majority of participants were recruited from a registry maintained by our research team. This may have introduced bias since participants had pre-existing relationships with the research team (e.g., we may have observed higher rates of engagement compared to a sample with no experience with our research team). Overall, participants were highly socially isolated during the study period, which may have increased feasibility and engagement rates. The intervention was intended to “nudge” participants toward building durable medication adherence habits. While results suggest that viral suppression was commonly sustained after receiving the lottery prize, a longer follow-up interval and exploration of the habit formation process would shed light on these potential mechanisms of action. We will also explore whether small prizes are as effective as larger prizes such as those provided in the present study (49).

### Conclusions

To achieve the goal of ending the HIV epidemic, a range of intervention approaches are needed to better serve the needs of African American/Black and Latino PWH, including lower-touch interventions that reduce unnecessary burdens on the public health and health care systems (72, 73). In particular, on-going or intermittent interventions may be needed for PWH in high-risk contexts, who tend to discontinue HIV antiretroviral therapy in times of crisis. The relatively low-touch approach grounded in behavioral economics and motivational interviewing tested in the present study is sufficiently promising to warrant refinement and study in future research, including testing the individual components in the multiphase optimization strategy framework.

## Data availability statement

The raw data supporting the conclusions of this article will be made available by the authors, without undue reservation.

## Ethics statement

The studies involving human participants were reviewed and approved by University Committee on Activities Involving Human Subjects at New York University. The patients/participants provided their written informed consent to participate in this study.

## Author contributions

MG obtained funding for the study, conceptualized the study, and led the effort to write the manuscript. SS was the project director and made important methodological contributions to the study, and served on the interpretive community. SL was a co-investigator and created the intervention approach used in the study. CC was a co-investigator and the study's principal statistician. SC assisted with study implementation, analysis of qualitative data, and also served on the interpretive community that interpreted and integrated findings. RF was the principal qualitative analyst and led the interpretive community that interpreted and integrated findings. KK assisted with study implementation and project direction. CD was a study co-investigator. KI and EP assisted with study implementation and served on the interpretive community that interpreted and integrated findings. All authors contributed to the article and approved the submitted version.

## Funding

This study was funded by the New York University Research Challenge Fund and the New York University Mega-grants Initiative in the Provost's Office, and supported by the NIDA-funded Center for Drug Use and HIV Research (CDUHR) at the NYU School of Global Public Health (P30DA011041; Holly Hagan, Ph.D., Principal Investigator. The NYU Silver School of Social Work provided valuable support throughout the study.

## Conflict of interest

Author RF was employed by Independent Consultant. The remaining authors declare that the research was conducted in the absence of any commercial or financial relationships that could be construed as a potential conflict of interest.

## Publisher's note

All claims expressed in this article are solely those of the authors and do not necessarily represent those of their affiliated organizations, or those of the publisher, the editors and the reviewers. Any product that may be evaluated in this article, or claim that may be made by its manufacturer, is not guaranteed or endorsed by the publisher.
