# Supplementary material for: Behavioral intervention grounded in motivational interviewing and behavioral economics shows promise with Black and English-speaking Latino persons living with HIV with unsuppressed HIV viral load in New York City: A mixed methods pilot study
Source: Front Public Health. 2022 Sep 15;10:916224. doi: 10.3389/fpubh.2022.916224 (PMC9522600; doi:10.3389/fpubh.2022.916224)
Supplement: Supplementary file 1 [file Data_Sheet_1.docx]

| **Appendix A: Supplemental materials – Text messages and Quiz Questions used in SCAP** | | | |
| --- | --- | --- | --- |
| **WEEK #** | **TEXT MESSAGE INFORMATION STATEMENT** | **TRUE/FALSE QUIZ QUESTION** | **ANSWER** |
| 1 | Hi [NAME]. This is your first health text message from SCAP! Did you know regular exercise (walking, running, biking, swimming, dancing, yoga, etc.) can help to maintain health and wellbeing? More info here: [URL redacted] | **** Regular exercise (walking, dancing, yoga, etc.) can help to maintain health and wellbeing. **** Press 1 for true, Press 2 for false. Respond to the text message to earn points and prizes. | TRUE |
| 2 | Hello, [NAME]. It’s your weekly SCAP text message! Did you know that if your HIV viral load goes down when you start taking HIV medication, it means that the medication is working well? Click the link for more info  [URL redacted] | **** If your HIV viral load goes down when you start taking HIV medication, it means that the medication is working well.****  Press 1 for true, Press 2 for false. Respond to the text to earn points and prizes. | TRUE |
| 3 | [NAME], hello from SCAP! Just a quick note to remind you that you can protect the ones you love – PrEP is there for them. [URL redacted] | **** Pre-exposure prophylaxis (PrEP) can prevent people from contracting HIV.**** Press 1 for true, Press 2 for false. Respond to the text to earn points and prizes. | TRUE |
| 4 | Hello, [NAME]. Today’s SCAP health text message is about smoking. HIV and smoking are a bad combination. Smoking hurts people living with HIV more than others. Here are some links to programs if you want to quit. [URL redacted] | ****Smoking does not hurt people living with HIV more than people who are not living with HIV.**** Press 1 for true, Press 2 for false. Respond to the text to earn points and prizes. | FALSE |
| 5 | Hi from SCAP, [NAME]. Did you ever take a break from HIV medication?  It’s okay! People can still benefit if they decide to start again. | ****People who have taken a long break from HIV medication can still benefit from medication if they decide to start again.**** Press 1 for true, Press 2 for false. Respond to the text to earn points and prizes. | TRUE |
| 6 | Hello, [NAME]. A lot of people in our SCAP community are worried about HIV medication side effects. Side effects are real, but today’s HIV medications are easier to take than ever. Click for more info [URL redacted] | ****Today’s HIV medications have fewer side effects than HIV medications in the past.**** Press 1 for true, Press 2 for false. Respond to the text to earn points and prizes. | TRUE |
| 7 | SCAP is thinking about you today, [NAME]! Here’s your health information: Today’s HIV medications are so effective, that if taken with high levels of adherence, people can live a long and healthy life | ****Today’s HIV medications are so effective, that if taken with high levels of adherence, most people can live a long and healthy life.**** Press 1 for true, Press 2 for false. Respond to the text to earn points and prizes. | TRUE |
| 8 | It’s your SCAP health text message day, [NAME]! Just a reminder that strong social relationships with friends and family can really help your physical and mental well-being. Click for more info [URL redacted] | ****Keeping strong, supportive relationships with friends and family does not help people be healthier overall.**** Press 1 for true, Press 2 for false. Respond to the text to earn points and prizes. | FALSE |
| 9 | [NAME], SCAP knows that stress is real. Feel stressed out? Try activities like meditation, acupuncture, and yoga. | ****There is not much you can do about stress, and activities like meditation, acupuncture, and yoga do not help.**** Press 1 for true, Press 2 for false. Respond to the text to earn points and prizes. | FALSE |
| 10 | Hello, [NAME]. SCAP is all about drinking a lot of water these days. Did you know staying hydrated can improve your energy and even your mood? | ****Drinking water is not part of staying healthy.**** Press 1 for true, Press 2 for false. Respond to the text to earn points and prizes. | FALSE |
| 11 | Hi from SCAP. Here’s your health text message for the week: One goal of taking HIV medication is to see your CD4 or T-cell count go up. | ****One goal of taking HIV medication is to see your CD4 or T-cell count go up.**** Press 1 for true, Press 2 for false. Respond to the text to earn points and prizes. | TRUE |
| 12 | Greetings, [NAME]. SCAP knows that Undetectable = Untransmittable! Did you know? If someone’s viral load has been undetectable for at least 6 months, there is no risk of transmitting HIV to someone else. [URL redacted] | ****Someone who has an undetectable HIV viral load level for six months or more has effectively no risk of transmitting HIV to someone else.**** Press 1 for true, Press 2 for false. Respond to the text to earn points and prizes. | TRUE |
| 13 | We at SCAP know that stopping smoking is no joke. But nicotine patches, nasal sprays, and gums are a safer option for people who smoke.  Click the link for more information. [URL redacted] | ****Nicotine patches are just as harmful as smoking regular cigarettes.**** Press 1 for true, Press 2 for false. Respond to the text to earn points and prizes. | FALSE |
| 14 | We wish stress was not a problem for our SCAP participants, but we know it often is. Feeling overwhelmed? Try taking deep breaths. That can reduce blood pressure and anxiety. [URL redacted] | **** When you are feeling overwhelmed, taking deep breaths can help you relax **** Press 1 for true, Press 2 for false. Respond to the text to earn points and prizes. | TRUE |
| 15 | Hello from SCAP. Need help remembering to take your medications? Using a pill box can help! Here are some additional tips to make taking medication easier. [URL redacted] | **Using a pill box will only make it harder to remember to take my medications**. Press 1 for true, Press 2 for false. Respond to the text to earn points and prizes. | FALSE |
| 16 | It’s time for your SCAP health text message! Not ready to start HIV medication? People can still benefit from going to a health care professional regularly | **People who choose not to take HIV medications can still benefit from attending regular health care appointments**. Press 1 for true, Press 2 for false. Respond to the text to earn points and prizes. | TRUE |
